# Generative Artificial Intelligence in Medical Education: Enhancing Critical Thinking or Undermining Cognitive Autonomy?

**DOI:** 10.2196/76340

**Published:** 2025-11-03

**Authors:** Juan S Izquierdo-Condoy, Marlon Arias-Intriago, Andrea Tello-De-la-Torre, Felix Busch, Esteban Ortiz-Prado

**Affiliations:** 1 One Health Research Group Universidad de Las Américas Quito Ecuador; 2 Institute for Diagnostic and Interventional Radiology School of Medicine and Health Technical University of Munich Munich Germany

**Keywords:** generative artificial intelligence, medical education, critical thinking, cognitive autonomy, curriculum innovation

## Abstract

Generative artificial intelligence (GenAI) enables the production of coherent and contextually relevant text by processing large-scale linguistic datasets. Tools such as ChatGPT, Gemini, Claude, and LLaMA are increasingly integrated into medical education, assisting students with a range of tasks, including clinical reasoning, literature review, scientific writing, and formative assessment. Although these tools offer significant advantages in terms of productivity, personalization, and cognitive support, their impact on critical thinking—a cornerstone of medical education—remains uncertain. The aim of this viewpoint paper is to critically assess the influence of GenAI on critical thinking within medical training, examining both its potential to enhance cognitive skills and the risks it poses to cognitive autonomy. Users have reported increased efficiency and improved linguistic output; however, concerns have also been raised regarding the risk of cognitive overreliance. Current evidence presents a mixed picture, indicating both improvements in learner engagement and potential drawbacks such as passivity or susceptibility to misinformation. Without curricular integration that prioritizes ethical use, prompt engineering, and critical evaluation, GenAI may compromise the cognitive autonomy of medical students. Conversely, when thoughtfully embedded into pedagogical frameworks, these tools can act as cognitive enhancers—supporting, rather than replacing, clinical reasoning. Medical education must adapt to ensure that future physicians engage with GenAI in a critical, ethical, and context-aware manner, especially in complex decision-making scenarios. This transformation demands not only technological fluency but also reflective practice and sustained oversight by faculty and academic institutions.

## Introduction

Critical reasoning is an essential component of medical training, enabling students to develop complex cognitive abilities that directly contribute to improved decision-making, deeper clinical insight, and safer patient care [1]. This mode of thinking extends beyond the mere recall or application of knowledge; it encompasses problem analysis, evidence evaluation, and the formulation of well-informed clinical judgments [2]. As such, critical reasoning is widely recognized as a cornerstone of high-quality medical education and professional practice [3].

Over the past few decades, technological advancements have profoundly transformed educational ecosystems, including those within medical schools [4]. The advent of the internet and metasearch engines revolutionized access to scientific information, democratizing medical education on a global scale [4-6]. More recently, a new paradigm shift has emerged with the rise of generative artificial intelligence (GenAI) [7]. Tools such as ChatGPT, Claude, DeepSeek, Gemini, Perplexity, LLaMA, and Google Med-PaLM are becoming increasingly embedded in the academic routines of medical students [8,9]. From supporting clinical reasoning and diagnostic processes to assisting with critical essay writing and literature reviews, these platforms are rapidly reshaping the landscape of medical education [10].

This manuscript does not present original data but offers a critical synthesis of current evidence and theoretical perspectives on the role of GenAI in medical education. It explores key issues such as the balance between technological assistance and reflective thinking, the role of faculty guidance, and the ethical implications of artificial intelligence (AI) use. Rather than providing definitive answers, this paper aims to inform pedagogical discussion and support future curricular development.

The aim of this paper is to critically examine the dual impact of GenAI on critical thinking in medical education, exploring both its potential to enhance cognitive skills and the risks it poses to cognitive autonomy.

## The Dual Impact of GenAI on Medical Education

GenAI is a subfield of artificial intelligence that employs advanced machine learning models to generate humanlike language [10]. Large language models such as ChatGPT, Gemini, Claude, LLaMA, and Mistral are built on transformer architectures that leverage self-attention mechanisms to evaluate the contextual relevance of words within a sequence [11]. Trained on massive datasets, these models can generate coherent, context-sensitive, and semantically rich responses across a wide range of tasks [12].

### Potential Cognitive Enhancer

GenAI tools have rapidly permeated educational ecosystems, often outpacing the development of appropriate regulations, curricular integration, and institutional guidelines. In medical education—a field characterized by dense curricula and high-performance pressure—these tools offer notable advantages: instant support for complex inquiries, rapid summarization of extensive content, clinical case simulations, and formative feedback [13]. Additionally, GenAI significantly enhances the presentation of assignments, essay quality, and research processes, streamlining academic productivity [14].

### Threat to Cognitive Autonomy

Despite its potential benefits, concerns persist regarding GenAI’s impact on students’ cognitive autonomy and critical thinking in medical education. Although some evidence points to enhancements in specific dimensions of cognition—particularly when GenAI is used within well-designed instructional frameworks—other studies highlight cognitive stagnation or even deterioration when AI tools are used without pedagogical scaffolding [15-17].

Cognitive autonomy refers to the learner’s capacity to make independent judgments, analyze information critically, and regulate decisions based on internal reasoning rather than external cues [18]. In medical education, fostering cognitive autonomy is essential to developing safe, competent professionals capable of making sound clinical decisions.

This concept aligns with the principles of self-regulated learning, defined as the extent to which students are metacognitively, motivationally, and behaviorally involved in their own learning process [19]. Self-regulated learning promotes autonomy, sustained engagement, and strategic thinking, all of which are central to critical reasoning in medical education.

However, uncritical or habitual reliance on GenAI tools may lead to cognitive offloading, wherein learners increasingly delegate analytical and creative tasks to external systems. This behavior may erode internal reasoning structures and reduce opportunities for deliberate practice and error-based learning—both core elements of clinical expertise. Without proper supervision or contextual understanding, students may also internalize biased, inaccurate, or hallucinated content [12,20,21]. Concerns extend beyond performance metrics. In a study of younger learners, Gerlich [22] found a significant negative correlation between frequent GenAI use and critical thinking scores, with cognitive offloading identified as a mediating factor [22].

These divergent findings suggest that the effectiveness of GenAI in supporting—or impairing—critical thinking among medical students is likely influenced by several variables, including tool design, task complexity, and the degree of faculty oversight [15,23].

Moreover, ethical risks such as plagiarism, dependency, or erosion of academic authorship may further compromise learners' sense of intellectual agency and accountability. In such cases, GenAI shifts from being a support tool to a potential threat to students' development as independent thinkers and professionals [24,25].

These conceptual risks are further supported by empirical and theoretical analyses (Table 1), which emphasize the negative impact of unsupervised GenAI use on learners’ cognitive engagement and higher-order reasoning skills.

**Table 1:** Representative studies illustrating the cognitive risks and benefits of generative artificial intelligence in educational settings.

Study	Study design, population	Key outcome	Relevance to our argument
Zhai et al [21]	Systematic review (18 studies)	Overreliance on AI^a^ correlated with reduced problem-solving ability (effect size = −0.41) and increased cognitive passivity in 78% of the studies	Supports cognitive autonomy risks with unsupervised use
Gonsalves [26]	Theoretical analysis	GenAI^b^ use disproportionately benefits lower-order Bloom’s skills (recall/comprehension) while weakening evaluation/creation without guided reflection	Explains why critical thinking erodes without design safeguards
Zhou et al [15]	Mixed methods, 325 graduate students	AI self-regulation (eg, bias-checking prompts) mediated 29% of the critical thinking gains. No gains occurred without this training	Validates digital literacy as prerequisite for AI integration
Gerlich [22]	Cross-sectional survey, 712 high school and early university students	Significant negative correlation between GenAI usage frequency and critical thinking scores; cognitive offloading was a mediating factor	Demonstrates how habitual GenAI use undermines critical thinking via cognitive offloading, especially in younger learners

^a^AI: artificial intelligence.

^b^GenAI: generative artificial intelligence.

## Empirical Evidence on the Cognitive Impact of GenAI in Medical Education

Although the current literature remains limited, recent empirical studies offer a more nuanced understanding of GenAI’s measurable cognitive effects in real-world medical education settings. Roos et al [27] conducted a large-scale study among German medical students, comparing the performance of GPT-4, Bing, and GPT-3.5 on items from the 2022 German Medical State Examinations. GPT-4 and Bing significantly outperformed students, achieving correct response rates of 88.1% and 86%, respectively, compared to 74.6% among the students. These results suggest considerable potential for AI-assisted preparation tools, although the study did not assess higher-order cognitive outcomes [27].

Regarding learning outcomes, Sakelaris et al [28] found no statistically significant differences in the exam scores between students who used GenAI tools (primarily ChatGPT) for studying and those who did not, indicating a limited direct impact on knowledge acquisition and academic performance in preclinical settings. Nonetheless, broader concerns regarding cognitive offloading and critical thinking persist.

In a separate study, Güvel et al [29] compared the performance of GenAI tools—including ChatGPT-4o, Gemini, and Claude—with that of human experts in generating case-based rational pharmacotherapy questions. Although AI-generated items showed comparable discrimination indices and correct answer rates (with Claude producing the highest proportion of error-free items—12 out of 20—and ChatGPT generating the fewest unusable items—5 out of 20), expert validation remained essential to eliminate flawed or unsuitable content.

More instructively, 2 recent randomized controlled trials clarify how instructional design mediates GenAI’s impact on critical thinking and engagement. Shalong et al [30] demonstrated that LearnGuide, a ChatGPT-based facilitator, improved scores on the Cornell Critical Thinking Test (+7.11; *P*<.001), self-directed learning (+4.15; *P*=.01), and cognitive flow in problem-based learning environments. These gains were sustained at a 14-week follow-up, highlighting the potential of structured, reflective GenAI use [30].

Conversely, Çiçek et al [31] found that ChatGPT-generated feedback did not significantly improve critical thinking and was inferior to expert-written feedback in managing complex diagnostic tasks. Nonetheless, students who later learned of the AI involvement exhibited heightened critical awareness, suggesting that transparency and reflection can positively shape digital literacy attitudes (*P*<.001).

Taken together, these findings underscore the context-dependent nature of GenAI’s impact and reinforce the importance of pedagogical scaffolding, expert supervision, and reflective engagement to preserve cognitive autonomy in medical education settings (Table 2).

**Table 2:** Comparative summary of generative artificial intelligence–assisted educational interventions and their impact on learning and critical thinking in medical education.

Study	Study design	Population	GenAI^a^ tool assessed	Main outcome	Implication
Roos et al [27], 2024	Comparative performance study	Germany medical students	GPT-4, Bing, GPT-3.5	GPT-4 and Bing outperformed students in knowledge recall	High potential for AI^b^-assisted test preparation
Sakelaris et al [28], 2024	Preclinical study (observational)	Medical students (n=38)	ChatGPT	No significant difference in exam scores with AI usage	Limited direct academic benefit in preclinical context
Güvel et al [29], 2025	Tool validation with expert comparison	Medical students (n=103)	ChatGPT-4o, Gemini, Claude	AI-generated items needed expert validation	AI tools can assist item generation but require expert oversight
Çiçek et al [31], 2024	RCT^c^	Medical students (n=129)	ChatGPT-generated feedback	Expert feedback outperformed AI in complex cases; AI raised critical awareness	Transparency and complexity shape AI's effectiveness
Shalong et al [30], 2024	RCT	Medical students (n=103)	LearnGuide (ChatGPT-based tool)	Improved self-directed learning, critical thinking, and engagement with AI tool	Structured integration enhances learning outcomes

^a^GenAI: generative artificial intelligence.

^b^AI: artificial intelligence.

^c^RCT: randomized clinical trial.

## Complementary Models: GenAI Versus Reasoning AI

In response to the cognitive limitations of GenAI, reasoning AI has emerged as a complementary paradigm [22]. While GenAI is known for its linguistic fluency and pattern recognition capabilities, reasoning AI systems are designed to emulate structured, goal-directed thinking by integrating logical inference, sequential problem-solving, and cognitive reasoning frameworks into their architecture [32]. These systems are typically built on neurosymbolic or neurological models that combine deep learning with symbolic reasoning, enabling them to construct traceable logical chains rather than merely predicting the next plausible output [33].

Although GenAI can generate broad lists of potential diagnoses, it often fails to establish step-by-step reasoning that accurately links clinical signs and laboratory findings. In contrast, a reasoning AI system could model diagnostic probabilities by using Bayesian networks, such as correlating hyperkalemia, peaked T waves, and angiotensin-converting enzyme inhibitor use to support a diagnosis of hyperkalemic αβ insufficiency [34].

Reasoning AI tools such as neurosymbolic workstations or diagnostic decision-support systems based on graphical models can be integrated into emerging educational approaches such as problem-based learning. In this context, they guide students through structured reasoning processes. For example, “Given this patient’s vital signs and laboratory values, propagate the data through an acid–base physiological model, then adjust the anion gap calculation step by step.” This scaffolding reinforces explicit clinical inference across a range of instructional scenarios [34,35].

Although GenAI may stimulate early stages of critical thinking by proposing hypotheses or generating broad explanations, reasoning AI requires disciplined, verifiable reasoning pathways—bringing learners closer to the structured deductive logic required in clinical practice. In curricular terms, an integrated model might begin with GenAI-driven brainstorming (eg, list all possible causes of chest pain) and progress to decision-analysis modules powered by reasoning AI (eg, apply a decision-tree model to differentiate myocardial infarction from pulmonary embolism) [35]. This would ultimately support a complementary AI integration model aimed at enhancing logical, structured reasoning in favor of more reflective, accurate clinical decision-making.

## Challenges to Preserving Critical Thinking in the AI Era

### Barriers to Safe Integration

Critical thinking is not simply a function of information access; it stems from confronting uncertainty, evaluating conflicting hypotheses, and making reasoned decisions often under pressure [36]. In medical training, these are not optional skills but core competencies [37,38]. The concern, then, is not that medical students are using GenAI as part of their learning process but that they may be delegating essential cognitive tasks to these tools without accompanying them with reflective processing [3].

This risk is especially acute in environments where students are not trained to critically engage with AI-generated outputs [21,23]. When AI becomes a substitute for expertise rather than a complement, students may forgo the processes that foster critical reasoning skills foundational to safe clinical practice [39]. If today’s and tomorrow’s medical students are not equipped to question information—regardless of its source—the integrity of their clinical judgment will be compromised [40,41].

### Principles for Ethical Implementation

Although some GenAI tools have shown promise in guided reasoning tasks, others still lack the contextual sensitivity and ethical awareness necessary to manage complex, atypical, or morally nuanced clinical scenarios [32,42]. Overreliance on such tools can result in the overestimation of their accuracy and reliability, particularly when design features are opaque or fail to include safeguards that alert users—such as medical students—to potential or common errors [26]. In a systematic review, Zhai et al [21] reported that excessive dependence on AI was associated with a significant reduction in problem-solving ability (effect size = −0.41) and cognitive passivity in 78% of the studies analyzed [21].

The ethical integration of GenAI into medical education demands a multidimensional approach that includes pedagogical scaffolding, algorithmic transparency, and robust institutional oversight. Đerić et al [43] highlight critical ethical domains for GenAI use in higher education, including copyright and authorship, transparency, user accountability, and academic integrity [43]. These areas must be explicitly incorporated into curriculum design and professional conduct training to prevent the normalization of ethically ambiguous practices.

Faculty should play an active role in cultivating students’ critical engagement with GenAI. This involves teaching students to scrutinize AI-generated outputs, identify algorithmic biases such as through prompt engineering tasks like “list the limitations of this AI-generated differential diagnosis,” and cross-check information against peer-reviewed sources [44]. Equally essential is the promotion of ethical literacy, especially among undergraduate medical students, whose understanding of academic responsibility and professional standards has been shown to be lower than that of faculty and researchers within higher education settings [43].

## Future Pathways: Toward AI-Augmented Medical Education

### Curriculum Innovation

**Figure 1:**
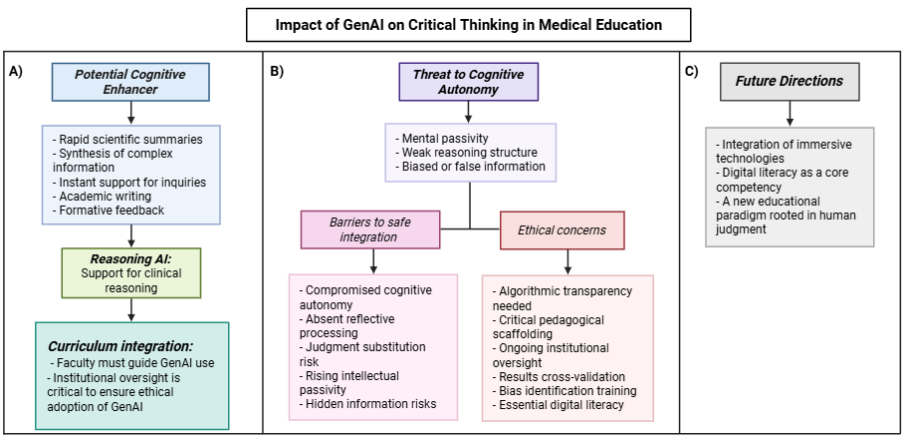
Conceptual model of the impact of GenAI on critical thinking in medical education. (A) Potential cognitive enhancer: GenAI can provide rapid scientific summaries, synthesis of complex information, instant support for inquiries, assistance in academic writing, and formative feedback. These applications can enhance reasoning AI by supporting clinical reasoning and, when guided by faculty and institutional oversight, contribute to curriculum integration. (B) Threat to cognitive autonomy: overreliance on GenAI may foster mental passivity, weak reasoning structures, and exposure to biased or false information. This creates barriers to safe integration, including compromised autonomy, absent reflective processing, judgment substitution, intellectual passivity, and hidden risks, and raises ethical concerns such as the need for algorithmic transparency, pedagogical scaffolding, institutional oversight, cross-validation, bias identification, and digital literacy. (C) Future directions: the safe and effective adoption of GenAI in medical education requires the integration of immersive technologies, development of digital literacy as a core competency, and promotion of a new educational paradigm firmly rooted in human judgment. AI: artificial intelligence; GenAI: generative artificial intelligence.

Although the evidence remains mixed regarding its influence on higher-order cognitive skills such as critical thinking, the integration of technology into modern curricula appears both inevitable and essential to prepare students for the demands of 21st-century education [45]. Faculty members must go beyond basic familiarity and proactively embed GenAI into their teaching strategies. This involves promoting active engagement with the tools [39,46,47], encouraging students to question outputs, identify biases, validate sources, and reflect critically on AI-generated information, such that integration must not diminish, but rather strengthen, cognitive autonomy [48]. This multidimensional relationship is summarized in Figure 1, which outlines the key variables that mediate whether GenAI enhances or undermines cognitive autonomy in medical education.

Universities bear the institutional responsibility of evaluating the quality, accuracy, and ethical implications of GenAI tools before their widespread adoption. As personalized tutors or virtual classroom assistants, GenAI systems can support adaptive learning paths and formative assessment. However, this requires clear transparency in model design, strong data governance protocols, and continuous oversight by academic staff to avoid misinformation or algorithmic bias [49]. Zhou et al [15] demonstrated that 29% of AI’s critical thinking benefits depend on self-regulation training—validating digital literacy as a curricular imperative.

### Emerging Synergies: GenAI + Immersive Technology

Crucially, the future of GenAI in medical education must also embrace the expanding potential of immersive technologies. Virtual learning environments, augmented reality, and extended reality platforms, when combined with GenAI, can simulate clinical encounters, anatomy labs, or public health scenarios—offering experiential, safe, and scalable training modalities. These immersive spaces enhance engagement, reduce learning gaps, and prepare students for complex decision-making under uncertainty [50,51].

To ensure ethical and effective use, digital literacy should become a foundational component of medical training [52]. This includes prompt engineering, AI output interpretation, critical appraisal of algorithmic content, and bias recognition. Without these competencies, students risk becoming passive consumers of AI output rather than critical users capable of navigating complex health information landscapes [53,54].

Ultimately, critical thinking will not emerge from an algorithmic echo chamber but through deliberate practice, intellectual curiosity, and a curriculum designed to blend human judgment with machine efficiency. The opportunity lies not in resisting AI integration but in designing an educational paradigm where GenAI enhances not replaces clinical reasoning and ethical decision-making.

## Limitations and Future Directions

This manuscript presents a theoretical perspective and does not include primary data collection, code generation, or statistical modeling. It is also important to note that the development of this manuscript did not follow a systematic search strategy, which introduces a potential risk of selection bias and the omission of relevant studies. Nevertheless, the analysis is grounded in a critical synthesis of publicly available scientific literature, drawing exclusively from peer-reviewed studies indexed in major academic databases such as PubMed, Scopus, and Web of Science, intentionally selected for their relevance and impact.

Another important limitation is that the cognitive effects of the GenAI tools analyzed are highly dependent on the educational contexts in which they were evaluated. Therefore, the conclusions should be interpreted with caution. The generalizability of the findings is constrained by the heterogeneity of learner populations, curricular frameworks, and levels of pedagogical oversight across the reviewed studies.

Consequently, we propose several future research directions. First, controlled comparative trials are needed to assess the differential impact of GenAI versus reasoning AI systems on the development of critical thinking. Second, it is essential to design and validate instruments capable of measuring cognitive autonomy in AI-mediated learning environments. Finally, ethical and digital literacy interventions should be developed and implemented for both students and educators, aiming to mitigate risks of overreliance and to promote reflective, context-sensitive use of these technologies in medical education.

## Conclusions

The emergence of GenAI in medical education represents a profound inflection point—rich in potential, yet fraught with risk. These tools can support overwhelmed learners and educators, personalize instruction, and facilitate the development of cognitive skills. However, they are not pedagogically neutral. Without intentional design and critical engagement, GenAI may erode the very attributes of clinical judgment, ethical reasoning, and intellectual autonomy that define competent physicians.

The future of medical education lies not in rejection or blind adoption, but in thoughtful, ethically grounded integration. This requires digital literacy training, faculty-mediated scaffolding, and curricular frameworks that reinforce reflective reasoning. GenAI should be viewed not as a threat or a panacea but as a catalyst that exposes both the strengths and vulnerabilities of the current educational models. The challenge ahead is to prepare physicians who are not only technologically fluent but also critically empowered. That—more than any algorithm—will shape the future of care.

## Abbreviations

AI: artificial intelligence
GenAI: generative artificial intelligence
